# Generating high-fidelity privacy-conscious synthetic patient data for causal effect estimation with multiple treatments

**DOI:** 10.3389/frai.2022.918813

**Published:** 2022-09-14

**Authors:** Jingpu Shi, Dong Wang, Gino Tesei, Beau Norgeot

**Affiliations:** Anthem AI, Palo Alto, CA, United States

**Keywords:** artificial intelligence, causal inference, electronic health records, observational data, treatment effects, potential outcomes, model validation, hypertension

## Abstract

In the past decade, there has been exponentially growing interest in the use of observational data collected as a part of routine healthcare practice to determine the effect of a treatment with causal inference models. Validation of these models, however, has been a challenge because the ground truth is unknown: only one treatment-outcome pair for each person can be observed. There have been multiple efforts to fill this void using synthetic data where the ground truth can be generated. However, to date, these datasets have been severely limited in their utility either by being modeled after small non-representative patient populations, being dissimilar to real target populations, or only providing known effects for two cohorts (treated vs. control). In this work, we produced a large-scale and realistic synthetic dataset that provides ground truth effects for over 10 hypertension treatments on blood pressure outcomes. The synthetic dataset was created by modeling a nationwide cohort of more than 580, 000 hypertension patient data including each person's multi-year history of diagnoses, medications, and laboratory values. We designed a data generation process by combining an adapted ADS-GAN model for fictitious patient information generation and a neural network for treatment outcome generation. Wasserstein distance of 0.35 demonstrates that our synthetic data follows a nearly identical joint distribution to the patient cohort used to generate the data. Patient privacy was a primary concern for this study; the ϵ-identifiability metric, which estimates the probability of actual patients being identified, is 0.008%, ensuring that our synthetic data cannot be used to identify any actual patients. To demonstrate its usage, we tested the bias in causal effect estimation of four well-established models using this dataset. The approach we used can be readily extended to other types of diseases in the clinical domain, and to datasets in other domains as well.

## 1. Introduction

In health care, studying the causal treatment effects on patients is critical to advance personalized medicine. Observing an association between a drug (exposure or treatment) and subsequent adverse or beneficial event (outcome) is not enough to claim that the treatment (or exposure) has a significant effect on the observed outcome. This is because of the existence of confounding variables, defined as factors that affect both the treatments and outcomes. Randomized controlled trials (RCTs) have been the gold standard for estimating causal relationships between intervention and outcome. However, RCTs are sometimes not feasible due to logistical, ethical, or financial considerations. Further, randomized experiments may not always be generalizable, due to the restricted population used in the experiments. In the past decade, observational data has become a viable alternative to RCTs to infer causal treatment effects due to both the increasingly available patient data captured in Electronic Health Records (EHRs) (Henry et al., 2016) and the remarkable advances of machine learning techniques and capabilities. Typically, EHRs capture potential confounding factors such as race, gender, geographic location, eventual proxies of social determinants of health, as well as medical characteristics such as comorbidities and laboratory results.

Many causal inference models have been proposed to estimate treatment effects from observational data. Validation of these models with realistic benchmarks, however, remains a fundamental challenge due to three reasons. First, the ground truth of treatment effects in a realistic setting is unknown. In real world, we can not compute the treatment effect by directly comparing the potential outcomes of different treatments because of the *fundamental problem of causal inference*: for a given patient and treatment, we can only observe the factual, defined as the patient outcome for the given treatment, but not the counterfactual, defined as the patient outcome if the treatment had been different. Second, legal and ethical issues around un-consented patient data and privacy created a significant barrier in accessing EHRs by the machine learning community. In order to mitigate the legal and ethical risks of sharing sensitive information, de-identification of patient records is a commonly used practice. However, previous work has shown that de-identification is not sufficient for avoiding re-identification through linkage with other identifiable datasets (Sweeney, 1997; Malin and Sweeney, 2004; Emam et al., 2011). Third, most publicly available datasets support binary treatments, while there has been growing literature developing techniques with multiple treatments in recent years (Lopez and Gutman, 2017).

To address these challenges, in this work we generated a large-scale and realistic patient dataset that mimics real patient data distributions, supports multiple treatments, and provides ground truth for the effects of these treatments. The datasets we generated are synthetic patients with hypertension modeled on a large nationwide cohort of patient data including their history of diagnoses, medications, and laboratory values. We designed a data generation process by adapting an Anonymization Through Data Synthesis Using Generative Adversarial Networks (ADS-GAN by Yoon et al., 2020) model for fictitious patient information generation and using a neural network for treatment outcome generation. The synthetic dataset demonstrates strong similarity to the original dataset as measured by the Wasserstein distance. In addition, we ensured that the original patients' privacy is preserved so that our dataset can be made available to the research community to evaluate causal inference models.

We demonstrated the use of the synthetic data by applying our dataset to evaluate four models: the inverse probability treatment weighting (IPTW) model (Rosenbaum and Rubin, 1983), the propensity matching model (Rosenbaum and Rubin, 1983), the propensity score stratification model (Rosenbaum and Rubin, 1983), and one model in the doubly robust family (Bang and Robins, 2005).

To our knowledge, this is the first large scale clinical dataset that mimics real data joint distributions with multiple treatments and known causal effects. Since hypertension is a condition affecting nearly half of adults in the United States (116 million, or 47%), our generated dataset can be directly used for clinical researchers to develop and evaluate their models for this important disease. The approach we used can be readily extended to other types of diseases in the clinical domain, and to datasets in other domains as well.

## 2. Materials and methods

### 2.1. Patient data and inclusion exclusion criteria

To make our synthetic data realistic, we generated the data based on a real-world patient database from a large insurance company in the United States. This database contains 5 billion insurance claims (diagnoses, procedures, and drug prescriptions or refills) and lab test results from 56.4 million patients who subscribed to the company's service within a 5-year time period between December 2014 and December 2020. From this database, we extracted a subset of patients affected by hypertension. Patients were included in the dataset if they had a medical claim indicating hypertension (ICD code I10, I11.9, I12.9, and I13.10) or treated with anti-hypertensive medications. We excluded patients from the dataset if they were age <18 or age >85, affected by white coat hypertension, secondary hypertension, malignant cancers, dementia, or were pregnant. After applying the above mentioned inclusion and exclusion criteria, we had about 1.6 million patients included in this study. We further excluded patients treated with a combination of drugs rather than a single drug. We then ranked the drugs by the number of patients treated with each drug, and only kept patients either treated with one of the 10 most popular drugs or not received any treatments at all. These filtering steps produced about 580, 000 patients in the study. The distribution of this dataset was then learned and used to generate synthetic patients, viewed as samples drawn from the learned distribution.

The patients' diagnoses and treatment history and how their conditions evolve over time were captured by trajectory data consisting of labs, diagnoses and their corresponding dates. For the convenience of data processing and analysis, we converted the trajectory data into tabular data with rows representing different patients (samples) and columns representing patient features (variables) including patient demographics, diagnoses, medications and labs. In Table 1, we list and briefly describe these 60 patient variables: 2 variables (F1) describing the systolic blood pressure before the treatment and the date it was measured, 2 variables (F2) describing the systolic blood pressure after the treatment and the date it was measured, 3 variables (F3) indicating current and prior drug usage and refill information, 4 variables (F4) describing patient basic information (age, gender, ethnicity), 30 variables (F5) indicating laboratory measurements, 2 variables (F6) indicating the presence or absence of comorbid conditions defined by the Charlson Comorbidity Index (Charlson et al., 1987), 15 variables (F7) describing the patient's zip code, the racial makeup and income levels in the patient's zip code tabulation area (ZCTA), 2 variables (F8) indicating meta information. The causal effects of anti-hypertensive drugs (current drugs of F3) on patient outcomes were measured as the difference between the first (F1) and second lab results (F2).

**Table 1:** Names, grouping, and descriptions of patient variables for hypertension dataset.

**Var. family**	**Var. names**	**Description**
F1	Date-, lab-	First lab result and date
F2	Date+, lab+	Second lab result and date
F3	Drugs, prior_drugs, last_refill	Drugs' info
F4	Age, gndr_cd, race_cd, ethncty_cd	Age/Gender/Ethnicity
F5	Lab measurement results and date	11 lab measurements and date
F6	Safety_morbs, morbs_prior	Current and previous comorbidities
F7	Zip_cd, total_pop, p_female, median_income etc	Zip code and related statistics
F8	Trajectory_index, mcid	Meta-information

### 2.2. Methods

To generate the synthetic data, we first generated the patient variables using an adapted ADS-GAN model, then generated the treatment outcomes using a neural network. Our approach can be conceptually decomposed into four steps described below.

#### 2.2.1. Step 1: Data preprocessing

Our goal was to generate the synthetic data from the patient data extracted in Section 2.1. In this step, we preprocessed the data and prepared it for subsequent steps. As described in Table 1, this patient dataset contains mixed data types including integers (e.g., age), floats (e.g., lab values), categorical values (e.g., drugs), and dates. Further, the values and dates of a lab test are missing for some patients if the lab test was not ordered by the doctors for these patients. We one-hot encoded the categorical variables and standardized the continuous variables so that all the variables were transformed into numerical values in the [0, 1] range. We then added a binary feature for each lab test to indicate missing lab values and dates. The resulting dataset has 200 features available per patient and we call it *the original dataset*, to be distinguished from the synthetic dataset.

#### 2.2.2. Step 2: Generation of observed variables using ADS-GAN

In this step, we generated synthetic patients characterized by the same variables as listed in Table 1. We wanted to achieve two goals: to make the synthetic data as realistic as possible and to make sure the probability of identifying any actual patients in the original dataset from the synthetic dataset is very low. We quantitatively define the identifiability in Definition 2 (Yoon et al., 2020), and the realisticity as the Wasserstein distance (Gulrajani et al., 2017) between the feature joint distribution of the synthetic dataset and that of the real dataset it is modeled after.

There is a trade-off between the identifiability and realisticity of the generated data. Frameworks like the Medical Generative Adversarial Network (MedGan, Choi et al., 2018) and Wasserstein Generative Adversarial Network and Gradient Penalty (WGAN-GP, Arjovsky et al., 2017) do not explicitly define and allow to control the identifiability levels. Therefore, we evaluated the generative models that allow to explicitly control such a trade-off, e.g., the ADS-GAN (Yoon et al., 2020), Private Aggregation of Teacher Ensembles Generative Adversarial Network (PATE-GAN, Jordon et al., 2019) and Diversity-promoting Generative Adversarial Network (DP-GAN, Xie et al., 2018). ADS-GAN proved to consistently outperform the others across the entire range of identifiability levels on both the MAGGIC (Meta-Analysis Global Group in Chronic Heart Failure) and the three UNOS (United Network for Organ Sharing) transplant datasets. It is also based on a measurable definition for identifiability. Another advantage of ADS-GAN is the use of Wasserstein distance to measure the similarity between two high dimensional joint distributions, which solves the limitation in the original GAN framework where the training of the generator and the discriminator is unstable (Yoon et al., 2020). We therefore selected ADS-GAN and adapted it by adding a contrastive term to its loss function to generate the patient variables in our study.

We denote the patient feature space by X. Let *X* be a *d*-dimensional random variable in X, subject to distribution *P*_*X*_. We use *d*-dimensional vector **x** to denote a generic realization of *X*, which is independently and randomly drawn from *P*_*X*_, where integer *d*> 1. The original dataset obtained in Section 2.2.1 is $D={\left\{{\text{x}}_{i}\right\}}_{i=1}^{N}$, where ${\text{x}}_{i}=\left(x_{i}^{\left(1\right)},x_{i}^{\left(2\right)}\ldots ,x_{i}^{\left(d\right)}\right)$, with $x_{i}^{\left(j\right)}\in X^{\left(j\right)}\subseteq \mathbb{R}$ representing the *j*-th feature of patient *i*. Here integer *N* is the number of samples and *d* is the number of features of each sample.

The goal of ADS-GAN is to produce a synthetic data set $\hat{D}=\left\{{\hat{x}}_{i}\right\}$, where each $\hat{\text{x}}\in {\mathbb{R}}^{d}$ is drawn from the distribution $P_{\hat{X}}$. Let *Z* be a random variable in space Z, and *z*~*P*_*Z*_ be the realizations of *Z* drawn from a multi-variate Gaussian distribution. We train a generator G:X×Z→X and a discriminator D:X→ℝ in an adversarial fashion: the generator *G* which produces synthetic patients ${\hat{\text{x}}}_{i}=G\left({\text{x}}_{i},z\right)$ ensures that the synthetic dataset $\hat{D}=\left\{{\hat{\text{x}}}_{i}\right\}$ is not too close to D as measured by the ϵ-identifiability defined below; on the other hand, the discriminator *D* which measures the distance between two distributions ensures that the distribution of generated patients $P_{\hat{X}}$ is indistinguishable from the distribution of real patients *P*_*X*_.

**Definition 1**. We define the weighted Euclidean distance *U*(**x**_*i*_, **x**_*j*_) between **x**_*i*_ and **x**_*j*_ as


$$U\left(x_{i},x_{j}\right)=\;\left\|w\left(x_{i}-x_{j}\right)\right\|,$$

where **w** = (*w*^(1)^, *w*^(2)^…, *w*^(*d*)^) is a *d*-dimensional weight vector.

To calculate *w*^*k*^ where 1 < = *k* < = *d*, we first calculate the discrete entropy of the *k*-th feature, i.e.,


$$\begin{array}{l} H\left(X^{\left(k\right)}\right)=-\underset{x^{\left(k\right)}\in X^{\left(k\right)}}{\sum }P\left(X^{\left(k\right)}=x^{\left(k\right)}\right)\log\left[P\left(X^{\left(k\right)}=x^{\left(k\right)}\right)\right] \end{array}$$

The weight *w*^*k*^ is then calculated as the inverse of *H*(*X*^(*k*)^). Since the theoretical range of entropy for a feature is [0, *log*(*N*)], the theoretical range for *w*^*k*^ is $\left[\frac{1}{log\left(N\right)},\infty \right)$. For our dataset, most feature weights are in range [0.25, 50]. In reality, if a patient can be re-identified, the re-identification is most likely through rare characteristics or medical conditions of a patient. Calculating the weight this way ensures that the rare features of a patient are given more weight, correctly reflecting the risk of re-identification associated with different features.

We now define *r*_*i*_ as


$$r_{i}=\;\underset{x_{j}\in D/x_{i}}{\min}U\left(x_{i},x_{j}\right),$$

where $D/{\text{x}}_{i}$ represents the dataset D without **x**_*i*_. From the definition, *r*_*i*_ is the distance between **x**_*i*_ and any other observation in D such that it is minimized. Similarly we define $\hat{r_{i}}$ as


$${\hat{r}}_{i}=\;\underset{{\hat{x}}_{j}\in \hat{D}}{\min}U\left(x_{i},{\hat{x}}_{j}\right).$$

**Definition 2**. The ϵ-identifiability of dataset *D* from $\hat{D}$ is defined as


(1) $$\epsilon =ℐ\left(D,\hat{D}\right)=\frac{1}{N}\sum_{i}\left[I\left(r_{i}>{\hat{r}}_{i}\right)\right],$$

where *I* is an indicator function.

We base the discriminator *D* on Wasserstein GAN with gradient penalty (Gulrajani et al., 2017) (WGAN-GP), which adopts Wasserstein distance between $P_{\hat{X}}$ and *P*_*X*_, and defines the loss $L_{D}$ for the discriminator *D* as


(2) $${ℒ}_{D}={𝔼}_{x\sim P_{X},\hat{x}\sim P_{\hat{X}}}\left[D\left(x\right)-D\left(\hat{x}\right)-\mu {\left({\left\|\nabla_{\tilde{x}}D\left(\tilde{x}\right)\right\|}_{2}-1\right)}^{2}\right]$$

where $\tilde{\text{x}}$ belongs to a random interpolation distribution between *P*_*X*_ and $P_{\hat{X}}$ and μ is a hyper-parameter that we set to 10 based on previous work (Gulrajani et al., 2017). We implemented both the generator and the discriminator using multi-layer perceptrons. To train the generator *G*, we need to compute the ϵ-identifiability by computing *r*_*i*_ and $\hat{r_{i}}$ for every sample, which is computationally expensive. To solve the problem, Yoon et al. (2020) made a simplifying assumption that *G*(**x**, *z*) is the closest data point to **x**. However, this assumption can be violated during the training of the network that maximizes the distance between *G*(**x**, *z*) and **x**. We here introduce a contrastive loss (triplet ranking loss, Schroff et al., 2015) term, which is defined as


(3) $$\begin{array}{ll} U_{con}\left(x,x^{\prime },z\right)= & \max\left(0,U\left(x,G\left(x,z\right)\right)-U\left(x^{\prime },G\left(x,z\right)\right)\right). \end{array}$$

Then, the final identifiability loss function ${ℒ}_{ℐ}$ is


(4) $$\begin{array}{ll} {ℒ}_{ℐ}= & {𝔼}_{x\sim P_{X},z\sim P_{z}}[-U(x,G(x,z))]+\beta {𝔼}_{x,x^{\prime }\sim P_{X}}[U_{con}(x,x^{\prime },z)]. \end{array}$$

Similar to Yoon et al. (2020), this loss function also assumes that *G*(**x**, *z*) is the closest data point to **x**. However, a penalty will be imposed if this assumption is violated when the generated sample *G*(**x**, *z*) is closer to **x**′, a randomly drawn sample from dataset D, than to **x**. The strength of the penalty term is controlled by β. In the final optimization problem, we minimize *G* and maximize *D* simultaneously, written as


(5) $$\begin{array}{ll} G^{*},D^{*}= & \arg\min_{G}\max_{D}\left[{ℒ}_{D}+\lambda L_{ℐ}\right] \end{array}$$

where λ is a hyper-parameter that controls the trade-off between the two objectives. Once trained, the adapted ADS-GAN model can be used to produce synthetic data set $\hat{D}$.

#### 2.2.3. Step 3: Data generation model and captured causal effects

A data generation model is needed to produce the potential outcomes for the synthetic data, i.e., the factuals and counterfactuals. Since the synthetic data is to be used to evaluate causal inference models, the ground truth of the causal effects needs to be known. Therefore, a causal mechanism needs to be explicitly built into the data generation process to ensure that the causal effects are indeed what cause the potential outcomes and can therefore serve as the ground truth to evaluate causal inference models. Although a completely predictive model can be used to produce the potential outcomes, it does not make the causal effects known and can not be used in such a data generation process. Many researchers used arbitrary data generation functions and arbitrary treatment effects to produce such synthetic data. For example, Schuler and Rose (2017) used a linear function as the data generation process and set the treatment effects arbitrarily. Such approaches are simple, but cannot produce synthetic outcomes that resemble real outcomes. In this work, we trained a neural network model on the original dataset to capture both the treatment effects with the network weights and the mapping from patient covariates to outcomes. We then used the learned mapping and treatment effects, along with the synthetic covariates as the network's inputs, to produce synthetic outcomes that resemble real outcomes. The captured treatment effects serve as the ground truth in the synthetic data when the data is used to evaluate causal inference models because the patient outcomes are generated from these causal effects.

Note that there is a distinction between the ground truth in the context of causal model evaluation and the true treatment effects in the real world. In our work, the captured effects are the ground truth in the synthetic data, but not necessarily the accurate true treatment effects of the treatments in the real world.

We partition the domain of observed patient variable *X* of *d* dimensions into the covariate domain $X_{C}\subseteq {\mathbb{R}}^{d_{c}}$, the treatment domain $X_{T}\subseteq {\mathbb{R}}^{d_{t}}$ and the outcome domain *X*_*o*_⊆ℝ, so that *d*≥*d*_*c*_+*d*_*t*_+1. The covariates are all the patient variables excluding drugs, prior drugs, zip code, and lab+. Treatments are the drugs. Outcome is the difference between lab+ and lab-. Each treatment **t**_*i*_∈*X*_*T*_ is one-hot encoded and represented by a *d*_*t*_ dimensional vector, where *d*_*t*_ is the number of treatments. In a cohort of *N* patients, for the *i*-th individual patient we use *Y*_*i*_, which is a scalar, to denote the potential outcome under treatment **t**_*i*_∈*X*_*T*_, and **x**_*ci*_ to denote the covariates of this patient. We assume that (*Y*_*i*_, **t**_*i*_, **x**_*ci*_)∈ℝ × *X*_*T*_×*X*_*C*_ are independently and identically distributed, which means that the potential outcomes for a patient are not impacted by the treatment status of other patients. We further assume that all the confounders are included in **x**_*c*_, and each patient has a none-zero chance of receiving any treatment. Therefore, the three fundamental assumptions for causal inference, SUTVA, unconfoundedness, and positivity, are satisfied (Rosenbaum and Rubin, 1983).

Following Lopez and Gutman (2017) and Shalit et al. (2017), given **x**_*ci*_ ∈ *X*_*C*_ and **t**_*i*_, **t**_0_ ∈ *X*_*T*_, where **t**_0_ is the zero-vector placebo, the individual-level treatment effect (ITE) of **t**_*i*_ can be defined as


$$\begin{array}{l} \tau_{t_{i}}\left(x_{c}{}_{i}\right):=𝔼\left[Y\left(t_{i}\right)-Y\left(t_{0}\right)|x_{c}{}_{i})\right]. \end{array}$$

Hence, the population average treatment effect for treatment **t**_*i*_ can be defined as


(6) $$\begin{array}{l} AT{𝔼}_{t_{i}}:=𝔼\left[Y\left(t_{i}\right)-Y\left(t_{0}\right)\right]=\int_{X_{C}}\tau_{t_{i}}\left(x_{c}\right)p\left(x_{c}\right)dx_{c}. \end{array}$$

The data generation process can be modeled as *Y* = Ω(**x**_*c*_, **t**), where $\Omega :X_{C}\times X_{T}\to X_{o}$. The true form of Ω is unknown and can be complicated. Here we make a simplifying assumption that the representation learned from the covariate domain is separated from the representation learned from the treatment domain. Specifically, let $\Phi :X_{C}\to R$ be a representation function and R be the representation space. We define $Q:ℛ\times X_{T}\to X_{o}$ so that Ω(**x**_*c*_, **t**) = *Q*(Φ(**x**_*c*_), **t**).

**Figure 1:**
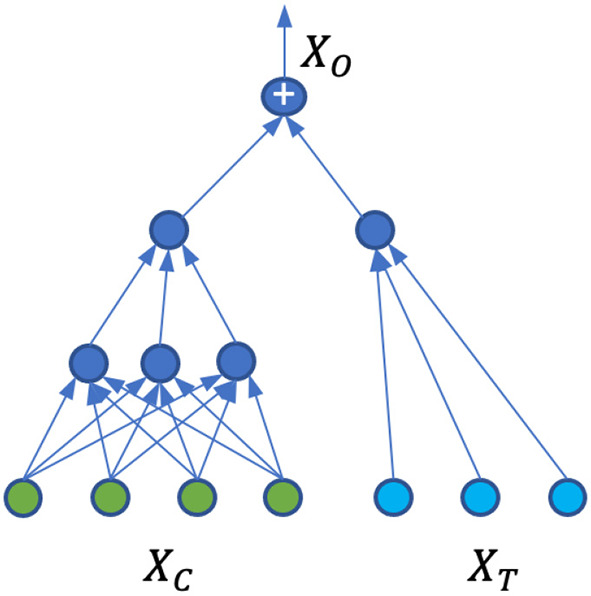
Neural network architecture for patient outcome generation and causal effect calculation.

With simplified Ω, we proposed a neural network architecture shown in Figure 1 that is able to capture Ω, Φ, and at the same time, calculate the treatment effects. For the covariate domain *X*_*C*_, the network is a fully connected feed-forward neural network with *Relu* as the activation function for all the neurons. For the treatment domain *X*_*T*_, the inputs are encoded treatments directly connected to a neuron with a linear activation. The loss function is the standard mean square error (MSE). A dropout is applied to all the layers and L2 regularization is applied to all the weights of the neural network.

The model Ω is trained on the original dataset described in Section 2.2.1, where we have one factual for each observation. Due to the separation of the covariate domain and treatment domain, and with the particular architecture of the ANN shown in Figure 1, the neural network weights for treatment connections can be interpreted as the causal treatment effects. Since there is no interaction between the covariates and treatments, the individual treatment effects and population average treatment effects are the same. Indeed, suppose **w** is the weight vector for treatment input **t**, then


$$Y(t_{i}|x_{c}{}_{i})=Q(\Phi (x_{c}{}_{i})+wt_{i}^{T}+e_{i}$$

where Φ(**x**_*ci*_) is the contribution to the neural network output from the covariate domain, $\text{w}{\text{t}}_{i}^{T}$ is the contribution from the treatment domain, and *e*_*i*_ is the error term. The outcome for the placebo **t**_0_ becomes,


$$Y(t_{0}|x_{c}{}_{i})=Q(\Phi (x_{c}{}_{i})+wt_{0}^{T}+e_{i}$$

According to Equation (6), the treatment effect is then


$$\tau_{t_{i}}=w(t_{i}^{T}-t_{0}^{T})=wt_{i}^{T}$$

Since **t**_*i*_ is a one-hot encoded vector, the treatment effect τ_**t**_*i*__ is just the weight of the neural network connection to the treatment given to this patient. One can similarly show that the weight is also the *ATE*_**t**_*i*__ in Equation 7.

#### 2.2.4. Step 4: Generation of factuals and counterfactuals

The domain of variables and all its partitions are the same for the real dataset D as for the synthetic dataset $\hat{D}={\left\{\hat{x_{i}}:\hat{x_{i}}=G\left(x_{i},z\right),x_{i}\in D,z\sim P_{Z}\right\}}_{i=1}^{N}$. Hence, the neural network trained on the original dataset in Step 2.2.3 can be fed with the synthetic patient variables generated in Step 2.2.2. The neural network outputs are served as the treatment outcomes for the synthetic data.

Once trained, this neural network is capable of generating all factual and counterfactual treatment outcomes for the synthetic data. For any synthetic patient with covariate $\hat{x_{cj}}\in X_{c}$, the potential outcome of any treatment *t*_*i*_∈*X*_*C*_ can be generated as $\hat{Y_{j}}\left(t_{i}\right)=\Omega \left(\hat{x_{cj}},t_{i}\right)=Q\left(\Phi \left(\hat{x_{cj}}\right),t_{i}\right).$ However, instead of generating the potential outcomes of all possible treatments in *X*_*T*_, in this work we only generated two potential outcomes for each patient: the factual outcome corresponding to the treatment produced by the ADS-GAN model, and the counterfactual outcome if the patient had not received any treatment. Note that we only produced one treatment in Section 2.2.2 for each synthetic patient with the ADS-GAN model, in order to preserve the treatment assignment mechanism learned from the original dataset, where each patient received only one treatment.

There is a distinction between the assumptions made in Section 2.2.3 in determining the treatment effects and the assumptions that our synthetic dataset actually satisfies. Specifically, our synthetic dataset satisfies the SUTVA and unconfoundedness assumption, as we did not model the interactions between patients and we provided all the patient variables in the dataset used to generate the outcomes. Whether the synthetic dataset satisfies the positivity assumption, however, depends on the original dataset because the patient assignment mechanism for the synthetic data is learned from the original dataset. The validity of this assumption can be checked by calculating the patients' propensity scores (Rosenbaum and Rubin, 1983). Violation of this assumption poses challenges to models that estimate causal effects based on propensity scores, such as the one proposed in Prescott et al. (2016).

### 2.3. Evaluations

To evaluate the quality of our synthetic dataset, we compared the joint data distributions between the original and synthetic datasets. We first calculated the Wasserstein distance (Villani, 2009) between the joint distribution of the synthetic data and that of the original data. The Wasserstein distance between two distributions ranges in [0, ∞] and can be interpreted as the optimal cost of transforming one distribution to the other (Villani, 2009). To put the calculated value in correct perspective, we measured the Wasserstein distance between the original dataset and a randomly generated dataset of the same dimensions. This serves as the baseline scenario. In addition, we randomly split the original dataset into two datasets and measured the Wasserstein distance between them, which is essentially the Wasserstein distance between the dataset and itself and serves as the best case scenario. We also visually compared the the joint distributions by plotting the heatmap of the two joint distributions side by side, and compared the marginal distributions of individual variables of the generated synthetic data with the corresponding ones from the original data.

Since the synthetic dataset we generated in this study is meant to be made public, patient privacy has to be preserved to ensure that no actual patients in the original dataset can be identified through the synthetic dataset. We calculated the ϵ-identifiability as defined in Definition 2 to evaluate whether patient privacy was addressed. We further calculated the ϵ-identifiability for the original data from a randomly generated dataset, which should be zero in theory but can be a small positive number due to a non-zero possibility of identifying a real patient from unrelated data. It serves as a reference of how small the ϵ-identifiability can possibly be. We then calculated the correlation matrix between the synthetic and original datasets to see how each variable of the synthetic data is correlated with every variable of the original data.

Finally, to demonstrate the usage of our dataset, we evaluated using our data the accuracy of causal effect estimate with four well-established models: the doubly robust (DR), the propensity score stratification, the propensity matching, and the inverse probability treatment weighting (IPTW) model. Doubly robust approaches adopt an outcome regression model to estimate the treatment outcome and a propensity model to estimate the probability of a patient being assigned to a treatment. In the DR model we tested, random forest is used as the outcome regression model. We used Microsoft DoWhy (Sharma and Kiciman, 2020) and EconML (Battocchi et al., 2019) causal inference packages for the implementation. When calculating the causal effect of a treatment, we removed all the counter-factuals from the dataset to prevent the problem from becoming trivial.

We adopted four metrics to evaluate the models: the Spearman's rank correlation coefficient to measure how well the models preserve the rank of the drugs by their treatment effects, the Kendall rank coefficient similar to Spearman's coefficient but based on concordant and discordant pairs, the Pearson correlation coefficient between the estimated effects and the ground truth, and finally the magnitude metric R-square (*R*^2^), measuring how much variance of the ground truth can be explained by the estimate. A comparison of the first three correlation metrics can be found in Coolen-Maturi and Elsayigh (2010), and a discussion of *R*^2^ can be found in Akossou and Palm (2013).

To estimate how these models perform in a real-world setting, we generated an additional dataset consisting of all patient variables of the original dataset and patient outcomes generated from the trained outcome neural network with patient variables and treatments from the original dataset as its inputs. We call this dataset *the hybrid dataset* because part of the data comes from the original dataset and part of the data is generated. We run the four causal inference models on both the synthetic dataset and the hybrid dataset and compared the results.

## 3. Results

This section reports the quality of our synthetic dataset. We found that there is strong similarity in both marginal and joint data distributions between the original and synthetic dataset, and that patient privacy is preserved.

### 3.1. Data similarity and patient identifiability

We first show how well the generated synthetic data preserves the joint distribution of the original data. We calculated the Wasserstein distance (Villani, 2009) between the joint distribution of the synthetic data and that of the original data to be 0.35, which is in the range (0.17, 8.6), where 0.17 is the Wasserstein distance in the best case scenario and 8.6 is the Wasserstein distance in the baseline scenario. We tried multiple random splits in the best case scenario and found that the Wasserstein distance varies very little with different splits.

**Figure 2:**
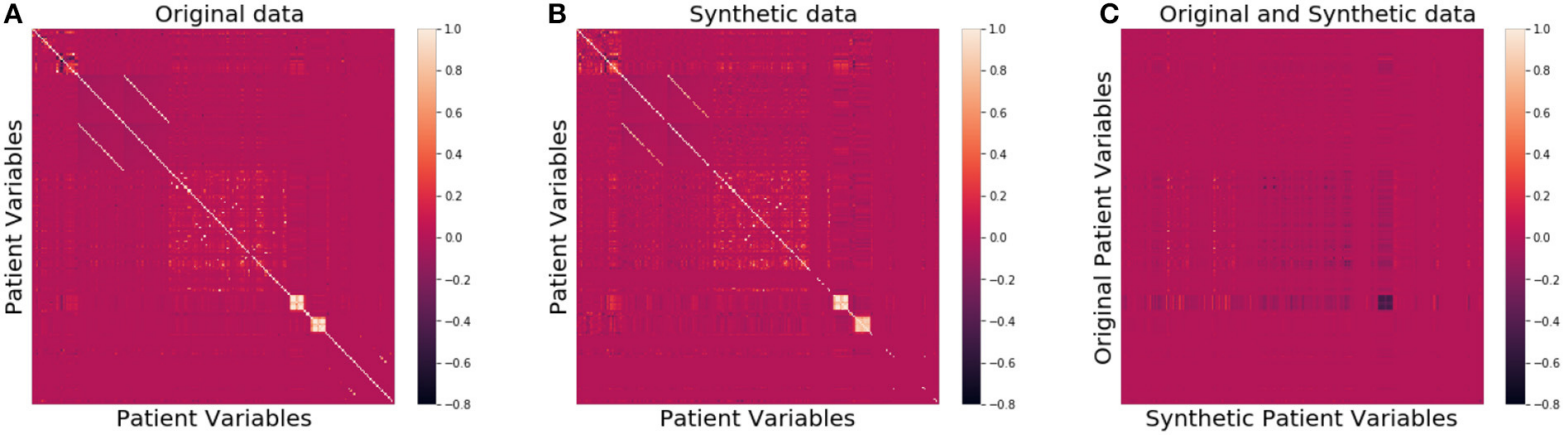
Heatmaps of correlation matrices of patient variables for the original **(A)**, synthetic **(B)**, and between original and synthetic data **(C)**, respectively.

We then compared the joint distributions visually. In Figures 2A,B, the correlation among all patient attributes in the original (synthetic) dataset is visualized by the heatmap on the left (right). In the heatmap, the brighter the color of a pixel is, the more correlated the two variables are with each other. The diagonal is the brightest in the map, as each pixel on the diagonal represents the correlation between a variable and itself. The two heatmaps show almost identical patterns, indicating the joint distribution of the original data is well preserved in the synthetic data.

**Figure 3:**
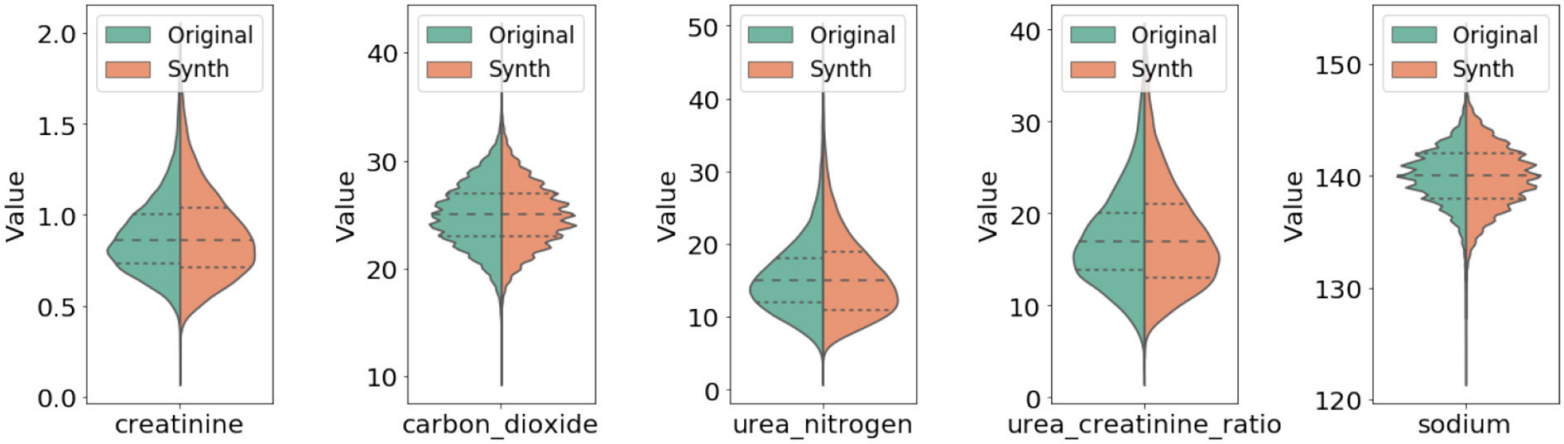
Comparison of marginal distribution of lab values between original and synthetic data. The three horizontal dotted lines in each violin plot from top to the bottom represent the third quartile, median, and the first quartile, respectively.

In Figure 3, we compared qualitatively the marginal distributions of individual variables of the generated synthetic data (orange) with the related ones from the original data (blue). The figure shows strong similarity between the original and synthetic dataset in both basic statistical summaries (e.g., median and quartiles) and overall shape of these distributions.

As far as patient privacy is concerned, all the synthetic samples in our dataset are conceptually drawn from a distribution, so no single piece of information about any actual patients is directly carried over to our dataset. We further calculated the ϵ-identifiability as defined in Definition 2 to be 0.008% from the synthetic dataset, and 0.0007% from the random dataset, indicating that the risk of any actual patient being identified from the synthetic dataset is extremely small. Figure 2C shows that the correlation between the variables of the original data and those of the synthetic data is very low, consistent with the small ϵ-identifiability value reported above.

### 3.2. Evaluate causal inference algorithms using the dataset

We run the four causal inference models described in Section 2.3 on both the hybrid and the synthetic datasets and report all the results in Tables 2, 3.

**Table 2:** Model evaluation results on hybrid dataset.

	**Spearman**	**Kendalltau**	**Correlation**	**R^2^ score**
Doubly robust—RF	1.00	1.00	1.00	0.76
Propensity stratification	0.96	0.91	0.97	−0.23
Propensity matching	0.94	0.82	0.90	−1.01
IPTW	−0.22	−0.16	−0.28	−845.88

**Table 3:** Model evaluation results on synthetic dataset.

	**Spearman**	**Kendalltau**	**Correlation**	**R^2^ score**
Doubly robust—RF	0.94	0.82	0.94	0.51
Propensity stratification	0.76	0.60	0.80	−0.47
Propensity matching	0.42	0.24	0.40	−4.54
IPTW	−0.35	−0.16	−0.42	−1565.35

The results on the hybrid dataset (Table 2) show that the evaluated algorithms performed very differently: the doubly robust model produced the best results and captured both the ranking and the magnitude of the drug effects; the propensity stratification and matching model captured the ranking of the drugs, but were not able to correctly calculate the magnitude of the drug effects. The IPTW model was not able to produce correct results on the ranking, nor on the magnitude, which was not surprising due to its significant bias if the propensity model is misspecified (Austin and Stuart, 2017). The results on the synthetic dataset (Table 3) show a similar pattern. The doubly robust model performed the best, followed by propensity stratification and matching. IPTW performed the worst. Investigating why some models outperform others on the two datasets is out of scope of this work. Here we show that the synthetic data preserves the relative performance of different models that would be achieved in a more realistic setting, represented by the hybrid dataset.

We reduced the size of the synthetic data and observed how the model evaluation results changed with smaller data sizes. When the size was reduced to 20% of the original size, the results were still similar to those obtained with the full dataset. When the size was below 20%, however, the standard deviation of the results started to increase significantly.

## 4. Discussion

There are certain limitations of our work. The inclusion and exclusion criteria applied to the data in this work may introduce selection bias. Our work was designed with a target trial in mind in which patients are recruited at an initial qualifying measurement and then followed up after treatment assignments. We believe this minimizes the impact of selection bias from conditioning on the inclusion and exclusion criteria in our original data. In this work, we produced one dataset for hypertension and evaluated four causal inference models. We leave it to future work to produce synthetic datasets for other diseases and evaluate and compare other causal inference models. Because hypertension affects almost half of adults in the United States, a synthetic dataset on hypertension is of significant value by itself. For simplicity, in this study we made the assumption that the covariate domain is separated from the treatment domain and did not consider treatment modifiers, i.e., interactions between treatments and patient variables, when producing treatment effects. Modeling treatment modifiers is an interesting and important topic which we plan to address in the future.

Our work is related to several existing works on publicly available databases, fictitious patient record creations, and data generation processes. First used in Hill (2011), the Infant Health and Development Program (IHDP) is a randomized controlled study designed to evaluate the effect of home visits from specialist doctors on the cognitive test scores of premature infants. The Jobs dataset by LaLonde (1986) is a benchmark used by the causal inference community, where the treatment is job training and the outcomes are income and employment status after training. The Twins dataset, originally used for evaluating causal inference in Louizos et al. (2017) and Yao et al. (2018), consists of samples from twin births in the U.S. between the years 1989 and 1991 provided in Almond et al. (2005). The Annual Atlantic Causal Inference Conference (ACIC) data challenge provides an opportunity to compare causal inference methodologies across a variety of data generation processes. In our work, we learned a data generation process from real-world patient data using a neural network, then used the learned network to generate patient outcomes.

Walonoski et al. (2018) generated synthetic EHRs based on publicly available information. The focus of their work was on generating the life cycle of a patient and how a disease evolves over time. Goncalves et al. (2020) evaluated three synthetic data generation models–probabilistic models, classification-based imputation models, and generative adversarial neural networks–in generating realistic EHR data. Tucker et al. (2020) used a Bayesian network model to generate synthetic data based on the Clinical Practice Research Datalink (CPRD) in the UK. Benaim et al. (2020) evaluated synthetic data produced from 5 contemporary studies using MDClone. Wang et al. (2021) proposed a framework to generate and evaluate synthetic health care data, and the key requirements of synthetic data for multiple purposes. Beaulieu-Jones et al. (2019) generated synthetic participants that resemble participants of the Systolic Blood Pressure Trial (SPRINT) trial. All of these works focus on data generation producing patient variables but without ground truth for causal effects. In contrast, the focus of our work was not only on generating patient variables, but on producing ground truth for causal effects as well.

To validate their models, many researchers such as Schuler and Rose (2017) created synthetic covariates and produced potential outcomes with a designed data generation process. Such datasets were not designed to approximate any real data distributions. Franklin et al. (2014) created a statistical framework for replicating the electronic healthcare claims data from an empirical cohort study and preserving the associations among patient variables. Neal et al. (2020) provided a benchmark for causal estimators by focusing on the simplest setting with no confounding, no selection bias, and no measurement error. All these works generated potential outcomes from covariates with known causal effects, but without any regard to patient privacy. We addressed the critical issue of patient privacy concerns so that our data can be made available for the research community to evaluate their models.

Some oversampling techniques such as the Synthetic Minority Oversampling Technique (SMOTE, Chawla et al., 2002) can be used to generate synthetic patients from real patients. These techniques do not explicitly address the patient privacy issue. Indeed, we implemented SMOTE and generated synthetic data with it. The ϵ-identifiability of the synthetic data generated this way was calculated to be 0.4%, much larger than the value 0.008% with our approach.

In summary, researchers have traditionally relied on labeled data, i.e., ground truth to validate machine learning models. Due to the fundamental problem of causal inference, however, the lack of realistic clinical data with ground truth makes it difficult to evaluate causal inference models. In this work, we produced a large-scale and realistic synthetic dataset by adapting an ADS-GAN model to generate patient variables and using a neural network to produce patient outcomes. The data we generated supports multiple treatments with known treatment effects. We demonstrated that this synthetic dataset preserves patient privacy and has strong similarity to the original dataset it is modeled after. We believe that it will facilitate the evaluation, understanding and improvement of causal inference models, especially with respect to how they perform in real-world scenarios.

## Data availability statement

The patient data are not publicly released due to HIPAA regulations and patient privacy. We report the link https://github.com/Jingpugit/synthetic-patient-data where our synthetic dataset in this study can be downloaded.

## Author contributions

BN conceived of the study and supervised the project. JS developed the methodology and designed the analyses. DW contributed to the software implementation. GT contributed to the algorithm design and literature review. All authors wrote and edited the manuscript. All authors contributed to the article and approved the submitted version.

## Funding

This study received funding from Anthem Inc. The funder was not involved in the study design, collection, analysis, interpretation of data, the writing of this article or the decision to submit it for publication.

## Conflict of interest

The authors declare that the research was conducted in the absence of any commercial or financial relationships that could be construed as a potential conflict of interest.

## Publisher's note

All claims expressed in this article are solely those of the authors and do not necessarily represent those of their affiliated organizations, or those of the publisher, the editors and the reviewers. Any product that may be evaluated in this article, or claim that may be made by its manufacturer, is not guaranteed or endorsed by the publisher.
